# Peripapillary Intrachoroidal Cavitation

**DOI:** 10.3390/jcm12144712

**Published:** 2023-07-16

**Authors:** Adèle Ehongo, Noélie Bacq

**Affiliations:** Department of Ophthalmology, Hôpital Erasme, Route de Lennik 808, 1070 Bruxelles, Belgium; noelie.bacq@hubruxelles.be

**Keywords:** myopia, peripapillary intrachoroidal cavitation, peripapillary staphyloma, gamma peripapillary atrophy, border tissue, optic nerve sheaths, biomechanics

## Abstract

Peripapillary intrachoroidal cavitation (PICC) is a yellow-orange lesion, located at the outer border of the myopic conus. First described as a localized detachment of the retinal pigment epithelium, its intrachoroidal location was later revealed, justifying its current name. PICC is related to other myopic complications such as posterior staphyloma, but its pathogenesis is not clear to date. Although it has been considered a benign condition, most eyes with PICC show visual field defects, which leads to diagnostic uncertainty as these deficits resemble those seen in glaucoma. Furthermore, eyes with PICC may develop macular detachment with retinoschisis. Finally, misdiagnosis of PICC as a metastatic choroidal tumor may lead to unnecessary and anxiety-inducing investigations. Advances in optical coherence tomography (OCT) imaging have improved the visualization of ocular structures, contributing to the understanding of PICC. Recently, high optic nerve sheath traction forces during eye movements in highly myopic eyes have been suggested as promoters of PICC, renewing interest around this condition. However, a review of PICC is still lacking. Therefore, we aimed to provide a concise yet comprehensive overview of the current state of the art, focusing on OCT illustrations, pathophysiology and potential future perspectives based on the biomechanics of the optic nerve.

## 1. Introduction

Peripapillary intrachoroidal cavitation (PICC) is a myopic complication of which the prevalence is expected to increase, due to the increasing prevalence of myopia.

PICC is a well-circumscribed yellow-orange lobular lesion, located at the outer border of the myopic conus [1]. Advances in optical coherence tomography (OCT) have revealed that PICC is a hyporeflective intrachoroidal thickening [2] with little or no deformation of the overlying plane of Bruch’s membrane [3]. It appears as a neural-based triangular choroidal thickening in OCT sections crossing the optic nerve (ON) head [3,4]. A discontinuity in the border tissue of the choroid is often associated with PICC [3].

Visual field (VF) defects are reported in up to 73.3% of PICCs [5]. These VF deficits are similar to those observed in glaucoma [5], constituting a cause of diagnostic uncertainty. Additionally, macular detachment with or without retinoschisis can complicate the prognosis of PICC [6,7,8,9,10,11]. Knowledge of the PICC enables avoiding misdiagnosis as a metastatic choroidal tumor which can lead to unnecessary and anxiety-inducing investigations [1]. 

PICC is related to other myopic complications, namely posterior staphyloma and myopic tilted disc [4]. It is more common in eyes with a higher maculopathy category [12].

While the pathogenesis of PICC has been hypothesized for years, recent findings in the biomechanics of the ON have reopened the debate. Indeed, several methods have demonstrated high traction forces exerted by the ON sheaths on the peripapillary region of myopic eyes during ocular movements [13,14,15]. These biomechanical findings renew interest in the pathogenesis of myopic peripapillary changes because these pulling forces have been suggested as promoters of PICC [16]. In this context, we performed a thorough review of PICC with emphasis on pathophysiology using OCT illustrations and we discussed potential future perspectives.

## 2. Epidemiology

PICC is found in 5–17% of highly myopic eyes [4,17]. Variability in study populations and study designs interferes with the prevalence range of PICC [4,17,18].

Shimada et al. first identified the PICC in the fundus and then performed OCT imaging [17]. However, more recent studies have revealed that only 46.7% to 53% of PICCs diagnosed on OCT are detected on fundus photos [4,19,20]. In addition, they searched for PICCs in the area under the ON head and would have missed restricted PICCs in other peripapillary parts [17]. These two aspects may explain the low prevalence of PICC in this series [17].

Some studies set the minimum age for inclusion at 50 years [4] while others also recruited younger subjects [17,21,22]. Knowing that the prevalence of PICC takes off at approximately the age of 30 [17,19], the inclusion of subjects aged at least 50 years [4] focuses on those most likely to have a PICC, thus explaining the higher prevalence of PICC in these cases. 

Choudhury et al. found that the prevalence of PICC was 2.2% in the whole myopic group and 22% in highly myopic eyes [18].

The mean age at diagnosis of PICC is approximately 50 years [1,2,3,5,17,21,22,23,24], ranging between 19 and 79 years [19,25]. Shimada et al. showed that the prevalence of PICC takes off at age 30 and is maintained over 70 years [17].

There is no gender predilection [1,17,22] and PICC can be unilateral (53% subjects in one series) or bilateral [1,3,19,22,23].

PICC is more common in highly myopic eyes. The reported range for axial length is between 25 and 32 mm [1,20] and the refractive error ranges from −6 D to −23 D [1,21]. However, PICC is also present in non-highly myopic eyes and even in non-myopic eyes [19,21,22]. Non-myopic or low myopic patients with PICC were significantly older than those with high myopia and PICC, highlighting the combined role of structural weakening and time [19].

PICCs do not induce loss of best corrected visual acuity, unless other myopic complications are associated [1,23]. However, VF defects are reported in 66% [23] to 73.3% of PICCs [5] and macular detachment can complicate a PICC [6,7,8,9,10,11]. 

Although there is no known association between PICC with intraocular pressure or systemic disease [4], an anecdotal case of acquired PICC secondary to intercalary membrane detachment has been reported in an eye with coloboma [26]. 

## 3. Clinical Investigations and Diagnosis of PICC

### 3.1. Fundoscopy and OCT

**Figure 1:**
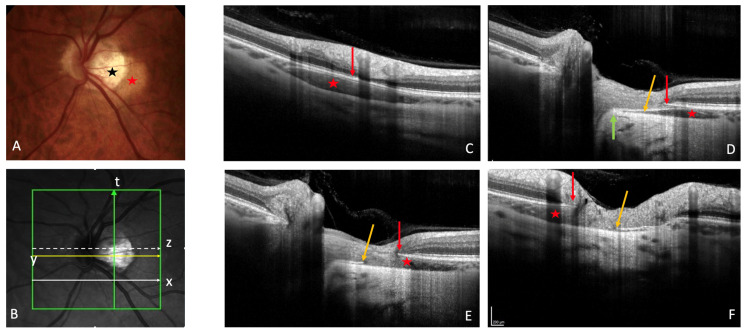
Peripapillary intrachoroidal cavitation (PICC). (**A**) Fundus picture. PICC is the yellow-orange lesion at the outer border of the myopic conus (black star) (**B**). Infrared image showing the location of sections (**C**) to (**F**). (**C**–**F**) PICC is the hyporeflective space behind the plane of Bruch’s membrane (BM). (**C**) Slice along the line x, below the optic nerve head (ONH). (**D**–**F**) Orange arrow = border tissue of the choroid (BT). (**D**) Section along the arrow y, through the ONH. The BT is continuous between the BM and the sclera (green arrow). (**E**) Section through the ONH, along the arrow z. The BT is discontinuous between the red and orange arrows. (**F**) Along the myopic conus (arrow t). The BT shows a discontinuity between the red and orange arrows. (**A**–**F**) Red star = PICC. (**C**–**F**) Red arrow = BM. The fundus picture was taken using VISUCAM^®^ non-mydriatic camera (PRO NM Carl Zeiss Meditec, Jena, Germany). The device used for OCT is the Spectral Domain OCT Spectralis^®^ OCT HRA-OCT, model S3300 (Heidelberg Engineering GmbH, Heidelberg Germany).

The yellow orange ophthalmoscopic appearance of PICC [1,3,4,17,19] (Figure 1A) is obvious only in 46.7% to 53% of OCT-detected PICCs [4,19,20]. Therefore, OCT is the recommended tool for the screening of PICC.

OCT reveals a spectrum of PICC appearances depending on the location and orientation of the section (Figure 1B). At the level of the peripapillary zone, it shows an aspect of hyporeflective intrachoroidal thickening [2] behind the plane of Bruch’s membrane (Figure 1C). If the section is through the ON, the PICC shows a triangular choroidal thickening (Figure 1D) with the base at the ON head. The well-demarcated border tissue of choroid (Figure 1D) may show discontinuity (Figure 1E,F).

The lesion is mainly located below the ON head [1,2,3,5,12,17,19,21,22,24]. However, other peripapillary areas may also be involved [4,10,12,17,22,24], and the PICC may even surround the entire ON head [17,19]. Therefore, Shimada subdivided it into three grades based on its overall circumference around the ON head [17].

Using spectral domain OCT or swept-source OCT, the diagnostic features of PICC are currently well established [2,3,21,22,27], obviating the need for other more invasive modalities such as fluorescein or indocyanine green angiography, the characteristics of which are summarized below.

### 3.2. Fluorescein Angiography

The sequence of fluorescein angiography shows early hypofluorescence followed by late hyperfluorescence without dye pooling in the area of PICC [1,2,17,22,23,28,29]. This angiographic sequence may be explained by structural choroidal changes. The early phase (hypofluorescence) results from disorganization, thinning, and loss of normal choroidal architecture while the late phase (hyperfluorescence) without dye pooling is related to the scleral impregnation by the dye, visible through the disorganized choroid [29]. 

### 3.3. Indocyanine Green Angiography

The area of PICC shows hypofluorescence throughout the indocyanine green angiography sequence [2,3,17,23]. This indicates slow or absent choroidal flow.

### 3.4. OCT-Angiography

**Figure 2:**
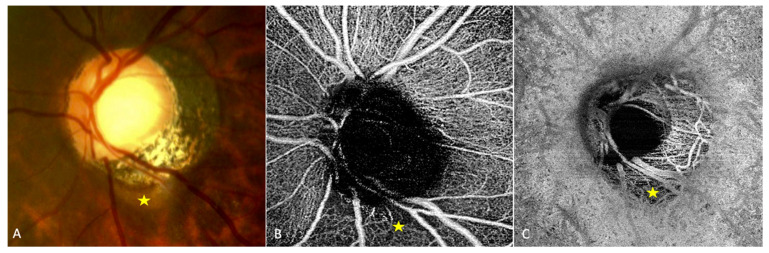
OCT-angiography of a peripapillary intrachoroidal cavitation (PICC). (**A**–**C**) Yellow star = PICC. (**A**) Fundus image with a PICC. (**B**) En-face OCT-A at the level of the superficial radial peripapillary capillary. Reduced vascular density at the area of PICC is observed. (**C**) En-face OCT-A at the level of choroid. Reduced vascular density is also seen at the area of PICC. The device used is the PLEX Elite^®^ 9000 SS OCTA (Carl Zeiss Meditec AG, Jena, Germany). A 6 × 6 mm field of view centered on the papilla. The fundus picture was taken using VISUCAM^®^ non-mydriatic camera (PRO NM Carl Zeiss Meditec, Jena, Germany). The device used for OCT-A is the PLEX Elite^®^ 9000 SS OCTA (Carl Zeiss Meditec AG, Jena, Germany). A 6 × 6 mm field of view centered on the papilla.

Analysis of highly myopic eyes showed that the vessel density of the radial peripapillary capillary network [30,31] (Figure 2B) and that of the ON head layers [30] were significantly reduced in eyes with than in those without PICC. Highly myopic and low myopic eyes also showed a reduced vessel density compared to non-myopic eyes [30].

**Figure 3:**
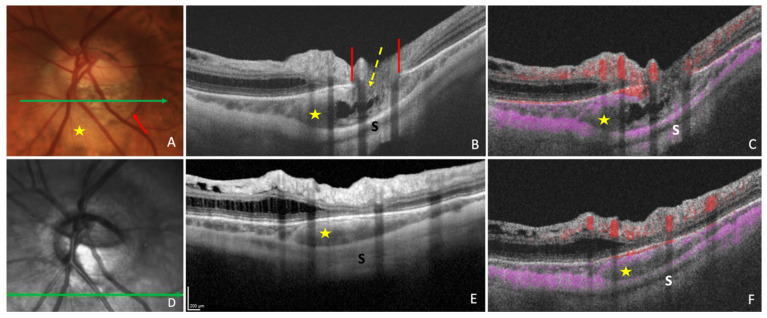
Optical coherence tomography (OCT) and OCT-angiography (OCT-A) in a case of peripapillary intrachoroidal cavitation (PICC). (**A**) Fundus image with a PICC at the outer border of the conus (red arrow). (**B**,**C**) Sections along the green arrow in (**A**). (**B**) OCT B-scan disclosing the PICC. Gamma peripapillary atrophy is between the two ends of the Bruch’s membrane (red lines). The retinal layers (dashed yellow arrow) herniate through them. (**C**) B-scan OCT-A showing the absence of signal inside the choroid. There is a signal (in pink) against Bruch’s membrane, corresponding to the choriocapillaris. Behind the intrachoroidal hyporeflective space, another signal corresponding to the sclera is perceived. The retinal vascular signal is red. (**D**) Infrared image with the green arrow indicating the location of sections. (**E**,**F**) The section is below the optic disc. (**F**) No signal is seen in the hyporeflective choroidal space between the sclera and the choriocapillaris. A signal is present at the level of choriocapillaris. PICC = yellow star. S = sclera. The fundus picture was taken using VISUCAM^®^ non-mydriatic camera (PRO NM Carl Zeiss Meditec, Jena, Germany). The device used for OCT and infrared image is the Spectral Domain OCT Spectralis^®^ HRA-OCT, model S3300 (Heidelberg Engineering GmbH, Heidelberg Germany). The device used for OCT-angiography is the Swept-source OCT Triton DRI Topcon corporation.

In a study of 47 glaucomatous eyes with PICC, Kim et al., using En-face OCT-A images at the choroidal layer, described two main vascular features. First, they noted a well demarcated homogeneous area of vessel density reduction matching the location of PICC [24] (Figure 2C). Second, they observed in 89.4% of cases, a choroidal microvasculature dropout (i.e., a focal sector with no visible choroidal and choriocapillary network). These similar patterns were previously reported in two case-reports of non-glaucomatous PICCs [32,33]. Using B scans of the OCT-A, Kim et al. showed that the PICC maintains the choriocapillary signal against the posterior surface of the Bruch’s membrane whereas it shows no signal in the intrachoroidal cavity [24] (Figure 3).

### 3.5. Other Modalities

Multimodal imaging [29,34,35] and three-dimensional reconstruction imaging [36] have been used in some cases of PICC, confirming the multiple facets of PICC. Azar et al. used fluorescein angiography and En-face OCT [29]; Chen et al. combined multicolor imaging, ocular B-scan ultrasonography, En-face OCT and enhanced depth imaging OCT [34]; Shen et al. used ocular ultrasonography A and B, fluorescein angiography and OCT [35]. 

By comparing conventional color fundus photography to multicolor imaging in a case of PICC, Venkatesh et al. found that the multicolor imaging would be less effective than conventional color fundus photography in diagnosing PICC in myopic eyes, which requires confirmation [37]. 

More recently, Fujimoto et al. developed a method of deep learning-based noise reduction and three-dimensional (3D) rendering of volumetric swept-source OCT images. Using this technology, they calculated the 3D volume parameter of PICC and assessed its value in detecting and understanding this condition. They found that the volume of PICC, as a new 3D parameter, reflects its influence on visual function [38].

## 4. Association of PICC with Other Myopia-Related Changes 

Although the pathophysiological mechanisms that underpin its OCT features remain to be understood, PICC is linked to several myopic changes. It is consistently located at the outer margin of the myopic conus [1,2,3,5,17]. It is correlated with myopic tilted disc and posterior staphyloma [4,39]. All cases of Freund’s series exhibited fundus complications of severe myopia [1].

### 4.1. Gamma Peripapillary Atrophy

PICC is always found at the outer margin of the conus [1,2,3,5,17,21]. Recent studies have shown that this region, also called γ-peripapillary atrophy (γPPA), is acquired [40,41], and results from stresses applied locally during myopic elongation of the eye [13,15,41]. It extends from the opening of the scleral canal to the edge of Bruch’s membrane. The disappearance of Bruch’s membrane in this area is confirmed by histological studies [42] and is visible on OCT [43]. 

### 4.2. Tilted Disc

Dai et al., by intraindividual inter-eye comparison of patients having unilateral PICC, showed that the eye with PICC was more spindle like because of a pronounced tilt [20].

The tilted disc is reported in 75% to 93.5% of cases of PICC, depending on the series [1,17]. A more recent study showed a prevalence of 100% [3]. 

A quantitative approach to estimate the magnitude of a disc tilt would be useful in assessing the true prevalence of tilted disc in the PICC [4,20,44]. 

### 4.3. Posterior Staphyloma

Posterior staphyloma is reported in 40.2% to 100% of PICC [19,45]. This discrepancy in prevalence may result from the study design and the diagnostic tools used [21,46]. Recently, wide-field OCT has shown its sensitivity to detect inconspicuous cases while also revealing the outermost border in case of very wide types of posterior staphylomas. It is therefore recommended for diagnosing posterior staphyloma [46,47]. 

Since tilted disc and posterior staphyloma are more common in myopic eyes [1,4,17], it is not surprising to find a higher prevalence of PICC in this refractive eye group. 

### 4.4. Others 

Myopia-related macular changes were found in 14% to 100% of eyes with PICC [1,19]. These include myopic macular degeneration, myopic choroidal neovascularization, foveoschisis, lacquer crack, patchy atrophy, retinal holes, and macular puckering [1,17,19,22,23]. 

## 5. Structural Changes in the Vicinity of the PICC

### 5.1. Peripapillary Atrophy

As mentioned above, each PICC is located at the outer margin of γPPA. The area of γPPA exhibits a posterior deformation of the sclera. Recent studies have suggested that the stress exerted in the peripapillary region during adduction may promote the emergence of γPPA. This stress is more marked at the location of γPPA [48].

### 5.2. Choroid

**Figure 4:**
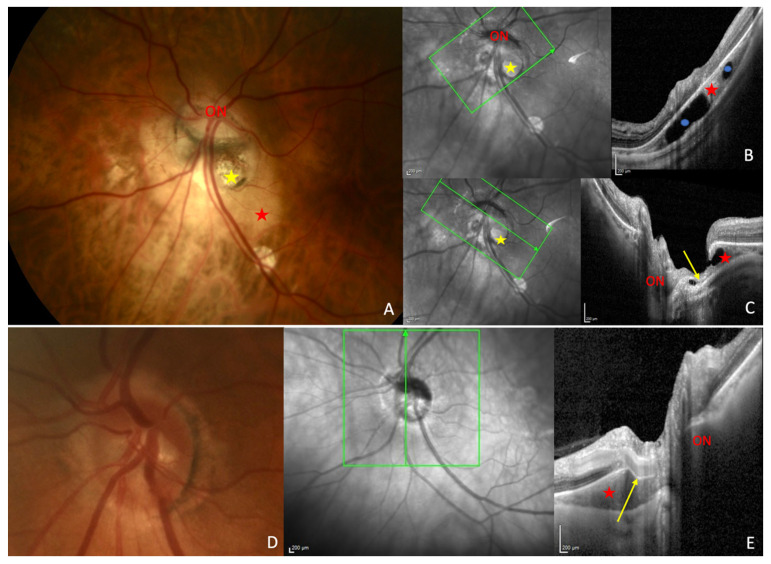
Structural choroidal findings in peripapillary intrachoroidal cavitation (PICC). (**A**) Fundus picture showing the yellow-orange aspect of PICC at the outer border of the myopic conus (yellow star). (**B**) Section along the green arrow in the corresponding infrared image. Intrachoroidal schisis (blue dots) are within the PICC cavity. (**C**) Section along the green arrow in the corresponding infrared image, through the full-thickness defect. This defect enables communication between the PICC and vitreous cavity. Nerve fibers (yellow arrow) herniate into the PICC cavity. (**D**) Fundus picture of the eye presented on OCT section. (**E**) The PICC is not apparent. (**E**) Section along the green arrow in the corresponding infrared image. The yellow arrow shows the bending of the temporal vessel in the PICC. ON = optic nerve. Red star = PICC. The fundus pictures were taken using VISUCAM^®^ non-mydriatic camera (PRO NM Carl Zeiss Meditec, Jena, Germany). The device used for OCT is the Spectral Domain OCT Spectralis^®^ HRA-OCT, model S3300 (Heidelberg Engineering GmbH, Heidelberg Germany).

Intrachoroidal hyporeflective cysts and hyporeflective hollows, disclosed by OCT, witness that in addition to choroidal thickening, PICC exhibits other structural changes inside the choroid [3,19,21,22,27]. These hyporeflective intrachoroidal cysts (Figure 4) were found in 19% to 39% of eyes with PICC [19,22]. Wei et al. described them as choroidal splitting or schisis adjacent to pocket-like spaces [22], while Yeh et al. reported intrachoroidal schisis characterized by intracavitary cleavage bands and fluid-like images [19].

Additionally, areas adjacent to intrachoroidal cysts show abnormal patterns of choroidal vessel [3]. Changes in the choroidal vascular signal were discussed in the OCT-A section above.

Considering the intrachoroidal hyperreflective line located in front of the hyporeflective space and separating this space from what appeared to be the residual initial choroid, Spaide et al. suggested that PICC was a suprachoroidal detachment [3]. This hypothesis is supported by a more recent study in which a suprachoroidal detachment was identified exclusively in cases of PICC [16].

Finally, Ehongo et al. studied the configuration of the posterior curvature of the choroid from the peripapillary polar regions to the opening of Bruch’s membrane using OCT. They highlighted a peculiar sequence of choroidal deformities associated with PICC, suggesting the role of mechanical forces in its pathogenesis. In particular, they described the presence of a posterior wedge deformity of the choroidal wall on the γPPA side, implying the existence of cross-forces at the level of the polar peripapillary regions [16].

### 5.3. Posterior Scleral Curvature

The sclera is deformed backwards in the PICC [3,17,21]. Spaide et al. have suggested that the choroidal thickening presented by PICC results from a posterior excursion of the sclera while the profile of the retinal pigment epithelium remains preserved [3].

A defect in the deep scleral layer allowing the exit of the inferior temporal vein into the extrascleral space associated with an unusual anatomy of the parapapillary region has been reported in a case of PICC [49].

### 5.4. Border Tissue of the Choroid

The border tissue is a fibro-astrocytic differentiation separating the nerve fibers from the surrounding structures (the choroid and the sclera) in the neural canal. It extends from the sclera at the level of lamina cribrosa to the Bruch’s membrane [50,51,52]. It is divided into two parts: the border tissue of choroid (Jacoby) lining the choroid, and the scleral border tissue (Elschnig) at the level of the sclera [50]. As the myopic conus extends during the myopic lengthening of the eye, the border tissue of choroid stretches [42]. A discontinuity in this structure has been disclosed using OCT [1,3,17,19,21,22,23] (Figure 1E,F). 

This discontinuity was first described as a cleft in the junction between the conus and the edge of the PICC in several studies since Freund’s paper [1,3,17,19,21,22,23] (Figure 4C). Its prevalence varies from 10% to 46.2% [1,22]. Through this full-thickness defect that enables communication between the PICC and vitreous cavity, nerve fibers (Figure 3B,C and Figure 4C) and retinal vessels (Figure 4E) have been shown to herniate into the PICC [1,3,17,25].

Discontinuity of the border tissue of the choroid would be caused by mechanical stresses exceeding its resistance and leading to its rupture. Several mechanisms have been mentioned; Toranzo et al. discussed the stress resulting from the posterior progression of peripapillary staphyloma [2]. Spaide et al. mentioned the stress related to the posterior deformation of the sclera within the thinned and fragile structures of the myopic conus [3]. Dai et al. suggested mechanical forces caused by papillary tilting [20]. Ehongo et al. discussed mechanical stress induced by the traction of the ON meninges during adduction [16].

**Figure 5:**
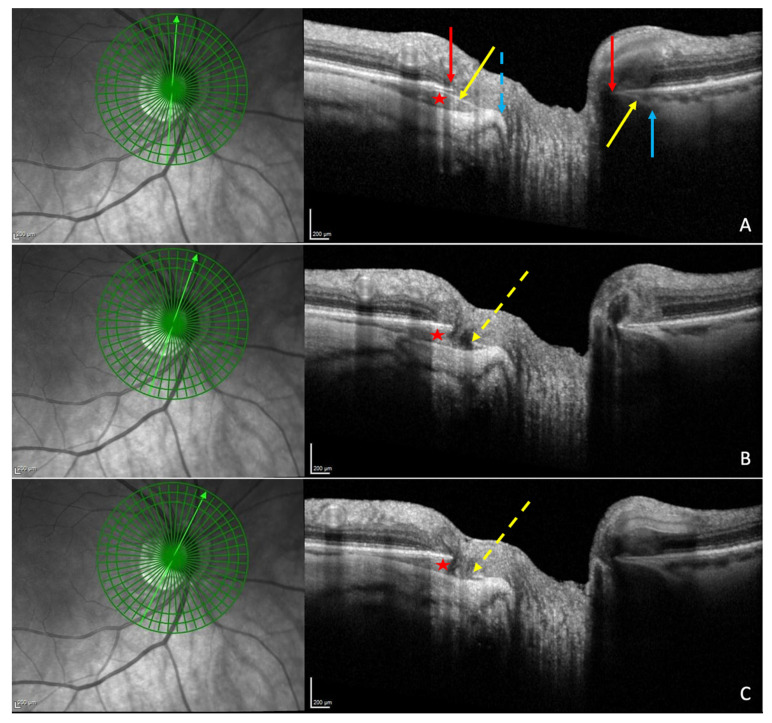
Discontinuity of the border tissue of choroid in a peripapillary intrachoroidal cavitation (PICC). (**A**) PICC presents as the hyporeflectivity behind the plane of Bruch’s membrane (red arrow). The border tissue of the choroid (yellow arrow) is the hyperreflective line between the Bruch’s membrane (red arrow) and the border of the scleral canal (dashed blue arrow). In the opposite side, it is between the red and blue arrows. In both cases, it is intact. (**B**,**C**) The border tissue of the choroid has a discontinuity (dashed yellow arrows) on the side with gamma peripapillary atrophy. (**A**–**C**) Red star = PICC. The device used is the Spectral Domain OCT Spectralis^®^ HRA-OCT, model S3300 (Heidelberg Engineering GmbH, Heidelberg Germany).

Recent versions of OCT allow to refine the diagnosis of this discontinuity by detecting it at the stage of a simple interruption of the hyperreflective line which characterizes choroidal border tissue in up to 25% of PICCs [3] (Figure 5).

### 5.5. Vessels

There is often a marked bending of the inferotemporal retinal vein (Figure 4E) into the steep excavation exhibited by the PICC at its junction with the conus [3,17,19,23]. This vessel sometimes disappears on part of its course in some PICCs with deep and steep excavations [3,17,19].

OCT-A features of PICC have been discussed above (Clinical investigation); Reduced vessel density in the En-face OCT-A at the choroidal level [24,32,33] is reported. Reduced vessel density in the radial peripapillary capillary plexus is also described [30] as well as that of the ON head layer [30]. Finally, the intrachoroidal cavity shows no vessel signal in the B scans of OCT-A [24] (Figure 3). 

An increase in visibility of peripapillary intrascleral vessels probably related to their dilation and scleral thinning has recently been demonstrated in the vicinity of PICC [53]. Further studies are warranted to clarify this finding.

## 6. Clinical Relevance of the PICC

Although the PICC has been considered a benign entity, it deserves special attention. A recent series showed that it was more common in the eyes of higher categories of myopic maculopathy [12]. Additionally, VF defects [5,23,24] and macular lesions (retinoschisis, macular detachment) can complicate the presence of a PICC [6,7,8,9,10,11].

### 6.1. Visual Field Defects and PICC

The main clinical significance of PICC is VF defects, which are reported [5,17,19,21,22,23,24,38,54] with a prevalence ranging from 37.5% to 73.3% [5,22]. They mimic glaucomatous VF defects, hence the concern about them [5,17]. 

A correlation has been found between the distribution of VF defects and the location of the PICC in some cases [5,23,38]. Analyzing glaucomatous eyes with PICC, Kim et al. Found that 98% of eyes had hemifield VF defects at the location corresponding to the hemispheric location of PICC [24]. 

The exact mechanism underlying VF defects in PICC is not established. It has been suggested that full-thickness defects, thinning, or disruption of nerve fibers at the PICC–conus junction may explain some of these VF defects [23].

Recently, Okuma et al. showed a correlation between the location of PICC and that of the reduced thickness of macular ganglion cell complex in 66.7% of cases, using OCT [5]. They also showed a correlation between the distribution of VF defects and PICC locations in 53.3% of cases, concluding that VF defects in PICC are similar to those in early glaucoma [5].

As a correlation between the location of the PICC and the distribution of VF defects is not found in all cases, this suggests that some of the VF defects found in eyes with PICC might result from myopic distortions. In Support of this hypothesis, Shimada et al. found VF defects in 23% of myopic eyes without PICC in their series [17]. 

Finally, a disc hemorrhage was found in a PICC without VF defect or reduction in the peripapillary nerve fiber layer [55]. In another case, VF defect could not be detected, even when testing the full-thickness defect exhibited by the PICC using microperimetry [25]. 

### 6.2. Macular Abnormalities and PICC

**Figure 6:**
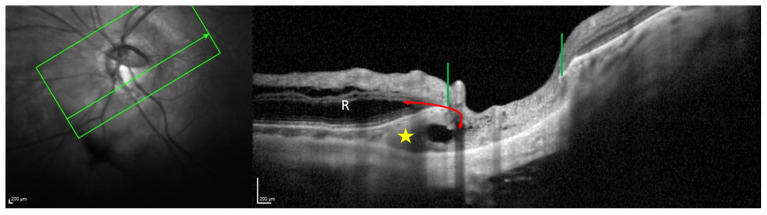
Illustration of a peripapillary intrachoroidal cavitations (PICC) presenting a retinoschisis. The section is along the green arrow in the infrared image. The PICC (yellow star) coexists with a huge retinoschisis (R). Nerve fiber layers herniate into the PICC cavity. The green lines indicate the ends of the Bruch’s membrane, allowing a communication between the PICC and the vitreous cavity. The appearance of a connection (double red arrow) between the PICC cavity and the retinoschisis would promote retinal detachment. The device used is the Spectral Domain OCT Spectralis^®^ HRA-OCT, model S3300 (Heidelberg Engineering GmbH, Heidelberg Germany).

From the first descriptions of PICC, a direct communication between the PICC and vitreous cavity at the junction between the conus and PICC has been reported [1,3,17] (Figure 6). Then, it was revealed that macular retinoschisis and macular detachment can complicate the presence of a PICC [6,7,8,9,10,11,12,56].

The pathophysiological mechanisms underlying macular detachment complicating a PICC involve two communications with the PICC cavity [6,7,8,10]. First, a connection to the vitreous through the full-thickness defect at the junction between the PICC and the conus, allowing vitreous fluid to access to the PICC cavity. Then, a second connection between the subretinal or intraretinal space and the PICC cavity (Figure 6). A narrow path connecting the PICC to the schisis has been documented in some cases [6,7,8]. It is suggested that this channel results from a rupture in the atrophic and dysplastic herniated retinal tissue, allowing vitreous fluid to track subretinally [6,7]. Vitreous traction [11] or peripapillary epiretinal membrane [8] have been implicated in some cases.

Although one case of self-resolving recurrent macular detachment and retinoschisis in one eye with PICC [56] has been reported, many cases of macular detachment with or without retinoschisis have undergone successful vitrectomy [8,10,11]. 

## 7. Differential Diagnosis—Natural History of Uncomplicated Cases of PICC

### 7.1. PICC and Differential Diagnosis

Nowadays, the diagnosis of PICC using the new versions of OCT (spectral domain-OCT and swept-source OCT) is well defined [3], thus making it possible to avoid unnecessary and more invasive and anxiety-inducing investigations. 

Additionally, for physicians unaware of the condition, the sequence of fluorescein angiography mentioned above helps differentiate PICC from other potentially confounding entities, namely pigment epithelial detachment, peripapillary choroidal neovascularization, metastatic lesion or central serous chorioretinopathy [2,17]. 

Finally, OCT-A is a non-invasive complementary diagnostic tool to PICC [24,32,33,34].

### 7.2. PICC and Glaucoma

PICC is asymptomatic [1,17] and has a good prognosis. However, as mentioned above, other vision-threatening myopic complications may accompany PICC [1,22], and VF defects are reported in PICC [5,17,19,21,22,23,24,38,54].

While the prevalence of PICC regarding the refraction is well known [4,17,18], the proportion of PICC in glaucomatous eyes remains to be analyzed. 

Importantly, glaucoma-like VF defects were reported in 66 to 73.3% of PICCs [5,17]. Interestingly, these deficits were found to have a location corresponding to the hemispheric location of PICC in 53.3% to 98.0% of cases [5,24]. Moreover, thinning of ganglion cell complex correlating the location of PICC was found in 66.7% (10/15) of eyes with PICC [5]. 

Hence, when PICC and related alterations are visible on the fundus or detected by OCT, this enables considering their potential link with VF defects found in the corresponding hemispheric location [5,24,54]. However, as PICC is detected on fundus in approximately 50% of cases [4,19,20], the presence of PICC should be considered in myopic eyes with glaucoma-like VF defects and should prompt to search for PICC by OCT. 

When it comes to treatment, Shimada et al. reported that of 22 eyes with VF defects, 18 were receiving glaucomatous medical treatments [17]; Wei et al. reported 6 eyes (37.5%) treated with drops for VF defects or structural alterations [22]. As VF defects in myopic eyes tend to have a slight progression under simple observation the benefit of anti-glaucoma drugs in PICC remains to be demonstrated [54]. 

Finally, the pathogenetic mechanisms between PICC and VF defects are still poorly understood.

### 7.3. Natural Course of PICC

In general, the PICC is stable. However, Freund et al. showed, in their series, a case of involution of the PICC in both eyes of the same patient over a follow-up of 15 years [1]. In these eyes, the PICC became smaller as γPPA widened towards it. 

The case reported by Toranzo et al. showed enlargement over 10 years [2]. Forte et al., using En-face OCT, observed PICC enlargement in one of six eyes in their series over an 18-month period [23]. Lee et al. reported an enlargement of the PICC over one year, followed by its shrinkage over another period of one year [55].

Formally, the condition being acquired and related to myopic complications, it is not surprising that it shows changes, albeit slow, over time, as the myopia and its structural modifications progress. 

Finally, the recently developed 3D volume calculation method [38] could prove promising for monitoring PICC volume over time and its influence on the VF.

## 8. Pathogenetic Hypotheses of PICC

So far, the pathogenesis of PICC is not established. Congenital, fluidic and mechanical hypotheses have been proposed. Similarities between PICC and the morphologic features at the border of the optic disc coloboma have been discussed [54]. All these pathogenetic hypotheses have weaknesses or shortcomings and are summarized below.

### 8.1. Congenital Hypothesis

Similarities between OCT characteristics of PICC and morphological features at the border of optic disc coloboma have been discussed [57]. 

Some authors have suggested that PICC could be an incomplete form of choroidal coloboma because it is mainly located in the inferior peripapillary region [1]. 

However, PICCs can extend widely around the papilla and some PICCs are restricted to the upper part of the peripapillary area which does not support this hypothesis [17]. Moreover, PICC is observed at approximately the age of 30 [17], suggesting that it is an acquired condition. Finally, its correlation with myopic tilted disc, γPPA and posterior staphyloma [4] suggests that it is another myopia-related condition.

### 8.2. Fluidic Considerations

Since the lesion is more common below the ON head, Freund et al. hypothesized that there might be gravitational displacement of subretinal fluid from the area of the ON [1]. This fluid would come either from the vitreous or from the optic canal [1].

Yeh et al. finding that subjects with non-myopic eyes and PICC were significantly older than those with myopic eyes and PICC, suggested that with aging, the transitional weakened tissue of the conus might present impaired resorption of fluids from the subretinal space, subarachnoid space, optic canal or vitreous cavity. The progressive and asymptomatic gravitation of these fluids would promote the formation of fluid pockets at the level of lower edge of the conus, hence the appearance of PICC [19].

However, this gravitational hypothesis does not explain why some PICCs are confined to the upper peripapillary part [4,17,22].

Many authors have suggested that the vitreous fluid enters the PICC through the retinal defect located at the PICC–conus transition zone [17,22,23]. In support of this hypothesis, Spaide et al. observed that PICCs with the opening against the vitreous showed a more prominent posterior bowing [3], while those without this opening showed a triangular thickening of the choroid with a neural base. 

Wei et al. suggested that after the rupture of the border tissue, the influx of vitreous fluid into the choroid would create a schisis or fluid pocket in the choroid [22]. They suggested that schisis and PICC might be different stages of the same phenomenon [22].

### 8.3. Mechanical Considerations

Wei et al. suggested that a complex of forces combining “posterior expansion force, vitreous tractional force and vitreous fluid dynamics would determine the size and shape of the PICC” [22]. However, vitreous traction has never been demonstrated in any uncomplicated case of PICC [3].

Lee et al. reported a case of PICC in a non-glaucomatous myopic eye accompanied by a large adjacent disc hemorrhage. A schisis in the prelaminar ON tissue was also noticed. At the one-year follow-up visit, the schisis and PICC had widened while the disc hemorrhage was still observed. The disc hemorrhage disappeared at the 2-year follow-up visit and the PICC showed shrinkage while the intraneural cyst was reduced. These changes suggest the presence of peripapillary mechanical forces [55].

Dai et al. showed by intraindividual inter-eye comparison that the eye with PICC was more tilted and more rotated, also suggesting the role of mechanical factors [20].

#### 8.3.1. PICC as a Complication of Peripapillary Staphyloma

Toranzo et al. suggested that the increased gap between Bruch’s membrane and the scleral planes resulting from thickening of the choroid as the posterior staphyloma progresses, stretches the border tissue which eventually ruptures. Secondarily, the choroid retracts from the ON margins, leading to a PICC [2].

Wei et al. added that adhesion of the retina and retinal pigment epithelium to the margin of the conus prevents the rupture from opening into the subretinal space [22]. 

#### 8.3.2. PICC as a Complication of Myopic Tilted Disc and Myopic Conus

As already mentioned, in the presence of γPPA (myopic conus), the sclera may be bowed posteriorly, giving rise to many suggestions.

Many authors have assumed that the posterior scleral bulge of the conus was favored by its weakening due to the absence of overlying structures [3,21]. From there, the force acting to deform the sclera depends on the author.

Shimada et al. hypothesized that during the process of γPPA extension the conus and the surrounding peripapillary area bow posteriorly. Mechanically, this induces a stretching of the tissues which then causes splitting of the neighboring intrachoroidal structures, with appearance of cysts inside the choroid. Then, the intrachoroidal cysts enlarge, coalesce, and end up in a large hyporeflective intrachoroidal space [21]. However, the flaw with this hypothesis is that the phenomenon of coalescence is not instantaneous. Therefore, hyporeflective intrachoroidal cysts, as precursors of PICC should also be found in the eyes of non-PICCs, which is not described in the literature. Further studies of longitudinal design should thus focus on these intrachoroidal cysts to clarify their impact on the pathophysiology of PICC.

Spaide et al. suggested that the driving force which is intraocular pressure against the wall of the eye induces the bulging of the weakened conus [3]. These authors explained that the stress/strain relationship ends with a more scleral deformation of the conus due to the absence or thinning of the tissues covering the sclera in this area. Subsequently, the posterior bowing of the sclera would itself favor the thickening of the neighboring choroid mainly in the inferior border of the ON. Finally, the widening of the choroid at the junction of the ON ends up breaking the border tissue, the latter promoting itself and secondarily the thickening of the choroid by allowing the entry of the vitreous fluid into the PICC [3].

Since the sclera of the conus deforms backwards, Forte et al. [23] hypothesized that the overlying structures are subjected to the same tendency. Unable to follow the strong posterior excursion of the sclera, they detach from choroid, thus creating the zone of cavitation inside the choroid and choroidal thickening.

#### 8.3.3. PICC as a Complication of the Optic Nerve Sheaths Traction

Toranzo et al. hypothesized that in posterior staphyloma, the choroidal border tissue ruptures when stretched by the increasing gap between the plane of the sclera and that of Bruch’s membrane [2]. The driving force that increases the gap between the sclera and the Bruch’s membrane is unknown. 

Beside their main hypothesis (intraocular pressure effect), Spaide et al. also opened the door to the possibility that in the tilted disc, unknown forces might be at play to bow the sclera backwards [3]. 

The question that remained open for years was: why in PICC does the sclera deform posteriorly while the overlying anterior structures remain undeformed? 

Recent studies on the biomechanics of the peripapillary region have shown by several methods that the ON sheaths exert strong tensile forces on the ON head and the peripapillary region [13,14,15] during eye movements. From the similarities between the tilted disc and intermittent distortions of the ON and peripapillary structures related to eye movements, some authors have suggested that tilted disc would result from remodeling and permanent fixation of these repetitive deformations [15,48,58,59]. Interestingly, a case of a preoperative round disc becoming oval after trabeculectomy has been reported [60]. The eye was highly myopic and had very high preoperative intraocular pressure. It seems that this elevated pressure counteracted the ovality of the disc. Therefore, the surgical reduction in eye pressure had promoted the return to the oval shape of the disc by altering the balance of forces acting on the ON head, suggesting that the phenomenon of tilted disc can be reversed to some extent. This constitutes an avenue to explore, because PICC and tilted disc are related. 

**Figure 7:**
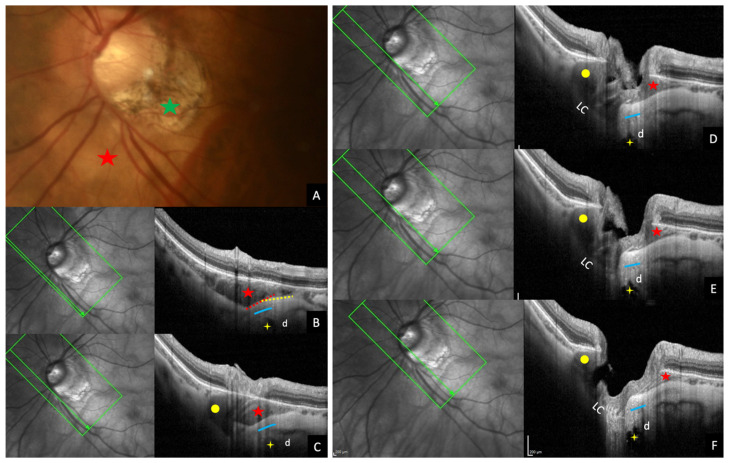
Traction of the dura mater in the pathogenesis of peripapillary intrachoroidal cavitation (PICC). (**A**) Fundus picture showing the yellow-orange aspect of PICC at the outer border of the myopic conus (green star). (**B**–**F**) Serial OCT sections towards the optic nerve head showing changes in the posterior curvature of the choroid and landmarks of PICC. (**B**) Wedge-shaped deformation of the posterior choroidal wall with the detachment of the supra-choroid. The dotted red and yellow lines outline the steepening of the scleral flange in front of the subarachnoid space. (**B**–**F**) The scleral flange is bowed backwards due to the traction of the dura mater. (**A**–**F**) Red star = PICC. (**B**–**F**) Yellow star = subarachnoid space. d = dura mater. (**C**–**F**) Blue line = scleral flange. Yellow dot = optic nerve. (**D**–**F**) LC = lamina cribrosa. The fundus picture was taken using VISUCAM^®^ non-mydriatic camera (PRO NM Carl Zeiss Meditec, Jena, Germany). The device used is the Spectral Domain OCT Spectralis^®^ HRA-OCT, model S3300 (Heidelberg Engineering GmbH, Heidelberg, Germany).

**Figure 8:**
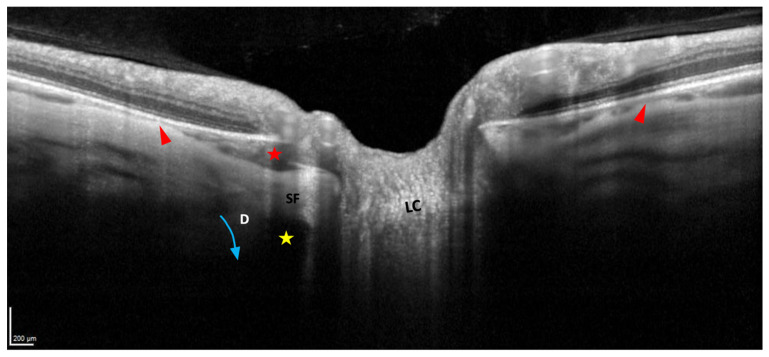
Landmarks of the peripapillary intrachoroidal cavitation (PICC). LC = lamina cribrosa. SF = scleral flange, the sclera between the dura and the pia mater. D = dura mater. PICC (red star) is in front of the subarachnoid space (yellow star). It is a suprachoroidal detachment. It is suggested to be promoted by a direct traction force (blue arrow) of the dura mater during eye movements. A tangential component of this traction force squeezes the choroid at the end of the posterior outpouching (red arrowheads). The device used is the Spectral Domain OCT Spectralis^®^ HRA-OCT, model S3300 (Heidelberg Engineering GmbH, Heidelberg Germany).

In this regard, very recently, Ehongo et al. confirmed that PICC is a suprachoroidal detachment (Figure 7A). They suggested that it was caused by the traction exerted by the ON sheaths on their scleral insertions [16]. They observed using serial OCT sections that the detection of the thickening of the choroid and the posterior bowing of the sclera, coincided with the detection of the scleral insertion of the dura mater (Figure 7A–F). The slope of the posterior bowing of the sclera steepened in front of the subarachnoid space, with the choroidal thickening ending in a suprachoroidal detachment (Figure 7A). They thus suggested that the posterior excursion of the sclera would result from the direct posterior traction exerted by the ON sheaths on the thinned and weakened myopic sclera (Figure 8). Due to this traction, the scleral flange (the part of the sclera between the margin of the ON and the dura mater) would recede while Bruch’s membrane, due to its structural rigidity would maintain its plane. This would lead to thickening of the choroid [16]. Further studies are warranted to confirm this hypothesis.

Again, relying on serial OCT sections, they observed that the convexity of the posterior choroidal wall was followed at its edge by an anterior elevation, suggesting that ON sheaths traction force would have two components [16]. The first which would act directly by protruding the sclera backwards. The second tangential component which would squeeze the choroid at the edge of the scleral convexity. This choroidal sequence would characterize a peripapillary staphyloma (Figure 8). They therefore suggested that peripapillary staphyloma would result from remodeling and permanent fixation of these repetitive deformations induced by pulling of the ON sheaths on the peripapillary sclera during eye movements [16]. These same repetitive traction forces have been suggested to promote the appearance of tilted disc [15,48,58,59].

## 9. Conclusions and Perspectives

The current increasing prevalence of myopia opens the way to that of high myopia and its complications, in particular posterior staphyloma, the presence of which classifies an eye in the group of pathologic myopia.

Peripapillary staphyloma and tilted disc are related to PICC. They have all been suggested to be promoted by the traction of the dura on its scleral attachment during eye movement. The link between these three complications of high myopia needs further investigation. Biomechanical and longitudinal studies are thus warranted to clarify mechanisms leading to them.

Tractions of the ON sheaths on the peripapillary region are exerted on all the eyes. On elongated myopic eyes, for obvious geometric reasons, these traction forces are more marked, promoting the occurrence of peripapillary myopic complications. However, these complications also occur, although less frequently in non-highly myopic eyes. Unknowns therefore remain as to whether the eyes undergoing these changes are more sensitive to ON sheaths traction force or whether this traction force is stronger in the eyes presenting these entities.

In particular, the influence of time and age justifies additional investigations since subjects with PICC and non-high myopia were found to be older than those with PICC and high myopia.

VF defects in PICC pose diagnostic difficulties with glaucoma (the prevalence of which is also increasing). Understanding the pathogenesis underpinning the occurrence of PICC and other peripapillary myopic complications will enable the development of strategies to slow or reverse their onset and associated VF defects.

A synopsis of publications on PICC is presented in the Appendix A.

## Data Availability

Not applicable.

## Appendix A

**Table A1:** A synopsis of publications on PICC.

Year Author [Ref]	Objective, Material, Methods	Relevant Results for PICC	Pathogenetic Clues	Strength
2003 Freund [1]	Objective: To describe a newly recognized fundus lesion in HM. Design: Retrospective study, case series (20 eyes of 15 patients included). Examinations: Fundus pictures and OCT (and FA)	Location: The inferior border of the myopic conus Associations: TD (75%), myopic conus (100%), PS (90%), fundus myopic changes (100%). Connection with vitreous at PICC–conus junction in 10% of PICC. Stable in the follow-up period (1–15 years) except for one patient with bilateral reduction in PICC.	Distinct complication of high myopia. Located at the inferior border of the myopic conus. Hypothesis: incomplete form of coloboma or gravitational accumulation of subretinal fluid coming from the optic disc/vitreous.	First description of PICC and recognition as a distinct fundus anomaly in highly myopic eye. Description of a PICC–vitreous communication in 10% cases.
2005 Toranzo [2]	Objective: To refine the PICC features Design: Observational case report Examinations: OCT-FA—ICG	The intrachoroidal location of PICC is revealed (with a preserved RPE plane and no detachment of the RPE). The size of PICC increased during the 10 years follow-up.	Mechanical hypothesis: progression of posterior staphyloma would stretch and break the BT, resulting in an intrachoroidal cavitation.	Intrachoroidal site of PICC revealed by OCT. Proposition of the current name (PICC) The RPE/BM plane and anterior structures is preserved.
2006 Shimada [17]	Objective: To study the prevalence and clinical characteristics of PICC. Analysis of VF in cases of PICC. Design: Prospective design (632 eyes with HM included). Comparison between PICC and a control group of HM. Examinations: OCT, FA, ICG and VF	Prevalence: 4.9% of PICC in HM (on fundus examination) Location: Inferior part in all cases Full-thickness defect at the conus-PICC junction in 10% of PICC. Marked posterior bowing of the sclera where PICC lies in 83.9% of cases. Association: TD: 93.5%. Myopic conus: 100%. PS: 64.3%. VFD: 71%. FA sequence: Early hypofluorescence and late hyperfluorescence without dye pooling.	The hypothesis of an incomplete form of coloboma unlikely: Lesion not confined to the inferior sector but can extent around the entire ON. No PICC in young patients: lesion probably acquired. Hypothesis: VFD could be caused by tissular distortion (TD and excavation of the myopic conus) and not by the PICC itself (could explain why the main location of PICC and VF defects do not always match).	VFD in 71% of PICC, no consistent correlation with PICC location. VFD significantly more frequent in PICC group than in control group. Communication with vitreous cavity in 10%. Marked bending of the inferotemporal vein in 83.9% of PICC. No PICC in subjects under 30 years old. Large series (632 eyes).
2007 Shimada [21]	Objective: To study peripapillary changes in eyes with HM using OCT. Design: Observational case series (127 eyes with HM included). Control group with low myopic and emmetropic eyes. Examinations: Fundus images, VF, OCT	Prevalence of PICC in HM on fundus examination: 9.4%. None in controls (emmetropic or low myopia). Prevalence of PICC in HM using OCT: 11%. PICC features on OCT: Intrachoroidal hyporeflective space showing multiple cystic spaces. Retinal full-thickness defects: In 7% of PICC. VFD in 64.3% of PICC and in 19.5% of HM without PICC.	Posterior excavation of conus related to PICC location. Hypothesis: Mechanical stress at peripapillary tissues associated with posterior excavation of the myopic conus could split the intrachoroidal structures and produce cystoid spaces leading to the PICC by coalescence. Description of retinal full-thickness defects leading to PICC–vitreous communication.	Glaucoma-like VFD in cases of PICC, and significantly more frequent than in the control group. OCT recommended for PICC diagnosis.
2008 Forte [23]	Objective: Evaluation of the thickness and lateral extent of PICC using en-face OCT. Design: Prospective case series: 6 eyes/3 patients. Examinations: En-face OCT, FA, ICG, VF	Retinal full-thickness defect in 2/6 eyes, allowing a PICC–vitreous communication. VFD in 4/4 eyes with anormal central fixation. VFD matching the PICC location in 3 of the 4 cases.	Hypothesis: Steep excavation of the myopic conus stretches the retina/RPE complex, causing a splitting of the choroid and a hyporeflective intrachoroidal space Gravitational accumulation of fluid from the vitreous cavity through a cleft at the border of the PICC could be an additional factor.	VFD in 4/4 eyes (Humphrey) The VFD matched the PICC location in 3 of the 4 cases.
2009 Wei [22]	Objective: Evaluation of OCT features and clinical aspects of PICC. Design: Observational case series (16 PICC diagnosed on fundus). Examinations: OCT, fundus, FA	Associations: Myopic conus: 100% Vitreous connection at the PICC–conus junction: 46.2% Intrachoroidal cystic spaces: 19% of PICC (intrachoroidal splitting/schisis without optical empty cavity). Inferior location in 94% of cases HM in all cases of PICC except one (moderate myopia).	Hypothesis: Vitreous fluid would gain access to the suprachoroidal space through the path caused by the breaking of the BT. Fluid accumulation induces a choroidal schisis and splitting of the choroidal structures. BT discontinuity is caused by the PS progression.	PICC and choroidal schisis would be different stages of the same pathologic spectrum. PICC are also in other locations than the inferior border of the optic nerve. History of glaucoma in 37.5% of cases
2009 Shimada [6]	Objective: To report a case of a macular retinal detachment related to a PICC. Design: Case report Examinations: OCT, fundus, FA	Retinal detachment in a highly myopic eye with PICC. No dye leakage on FA. A PICC–vitreous connection at the PICC–vitreous junction and a PICC–retinal detachment connection through subretinal space at the conus area.	Hypothesis: A retinal detachment can complicate a PICC through a subretinal connection with the full retinal thickness defect located at the PICC–conus margin.	PICC can be complicated by retinal detachment.
2011 Freund [27]	Objective: To study the PICC characteristics using OCT-EDI. Design: Case series Examinations: Fundus, OCT-EDI	On OCT-EDI, PICC is characterized by thickening of the choroid with or without hyporeflective “cavitation”.	PICC shows a choroidal thickening with variable hyporeflectivity.	PICC has several facets of presentations.
2012 Spaide [3]	Objective: To provide a pathogenic hypothesis based on anatomical characteristics of PICC. Design: Case series, 16 PICC included. Examinations: Fundus images, FA, ICG, SS-OCT and OCT-EDI	Associations: TD: 100%, myopic conus: 100%. Vitreous communication: 25% of cases (associated with more prominent cavitation). Posterior scleral bowing with normal retina-RPE plane in all PICCs. Dipping of inferotemporal vein into PICC at the conus edge.	Hypothesis: The posterior displacement of the sclera would be the primary cause of PICC. Rupture of BT by excessive stretching might lead to more prominent PICC.	PICC would start at suprachoroidal space. The radial section of PICC is triangular in the absence of a full-thickness defect and shows a more pronounced and rounded protrusion in its presence.
2012 Akimoto [56]	Objective: To report of a self-limited recurrent macular detachment associated with PICC. Design: Case report	Description of a PICC in a low myopic eye (−1 D) associated with a retinoschisis and a macular detachment.	A connection between PICC and retinoschisis may promote retinal detachment.	Macular detachment in PICC may occur in non-highly myopic eyes.
2013 Yeh [19]	Objective: Evaluation of the clinical features of peripapillary area associated with PICC. Design: Retrospective observational case series (inclusion of 122 PICC diagnosed on OCT, no control group). Examinations: OCT, VF, fundus pictures	Only 46.7% of PICC diagnosed on OCT are detected on fundus examination. Associations: PS: 40.2%, γPPA 98.4%, TD: 69.7%, VFD: 37.7% Maculopathy:14% Non-highly myopic patients with PICC are older (*p* < 0.05) Only 3 PICC in patients younger than 30. Fragmented aspect of the choroidal cavitation seen in 39%. Connection with vitreous cavity detected in 16% of PICC (26.4% in case of marked excavation of the myopic conus) Inferotemporal vein bent at the PICC border in 43.4%	Hypothesis: Possible impact of age in the pathogenesis of PICC (due to age-related reduced resorption of fluids). By gravitational effect, fluids would accumulate and form fluid pocket at the inferior border of the myopic conus. This could lead to the onset of PICC.	Less than 50% of PICC diagnosed on OCT are detected on the fundus examination. The presence of marked excavation of the myopic conus increases the incidence of PICC communication with the vitreous cavity. Implication of age in the pathogenesis of PICC.
2013 You [4]	Objective: Determine the prevalence, size, location of PICC and their associations. Design: Population-based study, (3468 patients included). Examinations: OCT-EDI, fundus pictures	PICC prevalence: 16.9% in HM (No PICC found in non-highly myopic eyes). Only 53% of PICC diagnosed on OCT were seen on fundus. Associations with TD and PS Location predominantly the inferior area No association with other ocular or systemic parameters.	Hypothesis: Distortions of the posterior fundus associated with PS and TD could be the primary cause of PICC in highly myopic eyes HM is not the primary cause of PICC (no association between PICC and AL on multivariate analysis).	Only 50% of PICCs are detected on fundus examination. Only TD and PS were associated with PICC. No association with other ocular or systemic parameters.
2013 Ohno [45]	Objective: Evaluation of ICC located temporal to the optic disc in highly myopic eyes. Design: Retrospective design, 125 highly myopic eyes included. Examinations: SS-OCT, FA	Prevalence of temporal ICC in highly myopic eyes: 12.8% Temporal ICCs are larger than inferior ones. Associations: myopic conus (100%), PS (100%) FA and OCT results are similar than those of PICC. Defects of the border tissue detected in some temporal ICC.	ICC can develop in temporal area only, without involving the inferior peripapillary area. Hypothesis: The separation of the temporal ICC develops at the suprachoroidal level (the entire thickness of the choroid remains attached to the RPE).	Consistent association between temporal ICC and both PS and myopic conus. Temporal ICC is a suprachoroidal lesion.
2014 Holak [57]	Comments on the Yeh et al. (2013) [19] article	Similarities exist between morphologic features at the border of optic disc coloboma and PICC.	They suggest exploring genetic, environmental, congenital impacts on the pathogenesis of PICC. Hypothesis: Cystoid spaces found in coloboma or PICC could be different stages of the same spectrum disease.	Mutations in cell adhesion molecules such as cadherin could promote coloboma and PICC formation?
2014 Yoshizawa [8]	Objective: To report a case of retinoschisis with macular detachment in a PICC treated by vitrectomy and outcome. Design: Case report Examinations: OCT Intervention: Vitrectomy	Retinoschisis with macular detachment. A connection between PICC and the schisis cavity was disclosed. Epiretinal membrane was adjacent to the PICC–conus connection. Complete regression of retinoschisis and closure of the PICC-retinoschisis channel.	The connection PICC-retinoschisis was suggested to be promoted by the traction on the PICC.	Good outcome of macular detachment associated by PICC when treated by vitrectomy.
2014 Rajagopal [7]	Objective: To report a case of macular detachment in a PICC. Design: Retrospective, case report Examinations: OCT, FA	A PICC–vitreous connection at the PICC–vitreous junction and a PICC–retinal detachment connection through subretinal space at the conus area.	/	Confirm that a connection between PICC and subretinal space may promote a macular detachment.
2014 Dai [49]	Objective: To describe the course of the inferotemporal vein into peripapillary region and to evaluate the characteristics of beta and gamma PPA. Design: Case report Examinations: Fundus pictures and OCT	Description of a case combining PICC, PPA and TD The inferotemporal vein disappeared in the Peripapillary area next to ON. On OCT, the detection of a scleral lamellar defect suggests an intrascleral or extra-scleral pathway of this vein.	/	Description of the abnormal course of the inferotemporal vein in an eye with PICC and PPA and TD.
2015 Chen [9]	Objective: To investigate clinical characteristics and treatment outcomes of macular detachment associated with PICC. Design: Retrospective case series	Depending on the case, a connection between the subretinal space and PICC or peripapillary area was found. Variable results with gas tamponade, topical carbonic inhibitors.	/	/
2015 Dai [20]	Objective: Intraindividual comparative study of ON morphology in unilateral PICC. Design: Hospital-based observational. Examinations: OCT-EDI, fundus pictures	Intraindividual comparison: eyes with PICC have lower ovality index, are more tilted, have a shorter vertical diameter and a shorter minimal diameter of ON compared to the contralateral eye. Only 53% of PICC are detected on fundus examination.	A shorter ovality index implies a rotation of the optic disc around the vertical or horizontal axis. Hypothesis: PICC is caused by the disruption of BT due to excessive disc tilting.	Optic disc is more tilted in eyes with PICC. 53% of PICC detected on OCT are not seen on fundus.
2015 Lee [55]	Objective: Description of a disc hemorrhage associated with a PICC in the absence of a glaucomatous neuropathy. Design: Case report Examinations: OCT-EDI, fundus pictures	A disc hemorrhage associated with PICC in a non-glaucomatous eye lasted more than a year. It showed a disappearance in conjunction with the reduction in size of the PICC and papillary schisis.	Peripapillary hemorrhages may be a manifestation of peripapillary stress. Hypothesis: Mechanical modifications due to PICC may alter the vessels and cause peripapillary hemorrhages without any glaucoma.	Impact of mechanical damage to peripapillary structures associated with PICC enlargement.
2015 Azar [29]	Objective: Multimodal imaging of PICC Design: Case report Examinations: SD-OCT, En-face OCT, ICG, FA, VF	En-face OCT combined with FA show an early hypo fluorescence resulting from the absence of choroidal tissue and a late staining resulting from the scleral impregnation.	/	Early hypofluorescence due to choroidal alteration. Late hyperfluorescence due to scleral impregnation
2015 Ando [10]	Objective: To report features of a macular detachment associated with PICC and the outcomes of vitrectomy. Design: Retrospective, case series (3 eyes). Examinations: Chart review. OCT	All 3 eyes were non highly myopic. No vitreous detachment observed in any case. Definite vitreous-PICC connection in 2 cases. Definite subretinal space-PICC connection in 2 cases. Vitrectomy resolved the macular detachment in all cases.	/	Macular detachment can complicate PICC even in non-highly myopic eyes.
2016 Jonas [59]	Objective: Comment (on Wang’s biomechanical study)	/	Suggestion that the stress exerted by the ON on its head during adduction movement could be part of the pathogenesis of the PICC.	Impact of ON biomechanics in the pathogenesis of PICC.
2016 Okuma [5]	Objective: Evaluation of the VF and the macular ganglion cell complex in PICC and evaluation of the similarities between these results and those typical of glaucomatous changes. Design: Retrospective, 16 eyes affected by PICC included. Examinations: OCT, VF	VF defects were detected in 73.3% of PICC. Good correlation between PICC location and VF defects distribution in 53.3% of cases. Thinning of ganglion cell complex was correlated with PICC location in 66.7%.	/	VF defects observed in 75% of PICC. PICC-VF defects correlation present in half of the with a PICC. Ganglion cell complex and VF defects found in PICC are very similar to those observed in early glaucoma.
2016 Kita [25]	Objective: Study of a PICC associated with a full thickness retinal defect in the papillo-macular bundle. Design: Case report Examinations: SS-OCT, VF	No visual field defect detected in the full-thickness defect located at the papillo-macular. Retina herniated in PICC may preserve some function despite the apparent retinal defect.	/	/
2017 Mazzaferro [33]	Objective: Evaluation of the characteristics of PICC on OCT-A. Design: Case report Examinations: OCT-A	Absence of choroidal and choriocapillary network in the choroidal cavitation. PICC associated with a PS and a TD.	/	Absence of any intrachoroidal vascular tissue in this case of PICC.
2017 Chen [30]	Objective: To study the peripapillary, ONH vasculature by OCT-A in highly myopic eyes with PICC. Design: Hospital-based cross-sectional study. Examinations: OCT-A	Highly myopic eyes show a lower peripapillary capillary vessel density than non-highly myopic eyes. The peripapillary vascular density is more reduced in PICC than non-PICC eyes (especially in temporal area).	/	The peripapillary vascular density is more reduced in PICC than non-PICC eyes (especially in temporal area).
2018 Chen [34]	Objective: Multimodal imaging of PICC associated with myopic sinkhole. Design: Case report Examinations: Fundus image, OCT-EDI US, VF	Description of a PICC associated with an inferotemporal sinkhole in the myopic conus. Presence of a cleft between the ICC and the vitreous cavity.	Hypothesis: myopic sinkhole could favour the flow of vitreous fluid through the suprachoroidal space and facilitate the formation of a PICC.	Possible role of the myopic sinkhole in the physiopathology of PICC.
2018 Choudhury [18]	Objective: Estimation of the prevalence of myopic degeneration in Chinese Americans. Design: Population-based cross-sectional study	1523 myopic Chinese included (<−0.5 D) Prevalence of ICC: 2.2% overall myopic eyes and in the 22% of HM eyes.	/	Population-based study (Chinese Americans).
2019 Venkatesh [53]	Objective: To study the prevalence and clinical characteristics of PICC. Design: Case series. Retrospective, non-interventional, comparative study. Examinations: fundus photography, OCT	Prevalence of ICC in highly myopic eyes: 55.8% (15.8% of PICC and 84.2% of macular ICC).	/	There is not a clear separation between two concepts in this study: PPA and patchy chorioretinal atrophy.
2019 Parlak [32]	Objective: Description of a case of PICC Design: Case report Examinations: Fundus photography, autofluorescence, OCT, OCT-A	Reduction in the vessel density at the level of the PICC with OCT-A.	/	PICC is characterised by a hypo signal on OCT-A.
2019 Shen [35]	Objective: Multimodal imaging of PICC Design: Case report Examinations: Fundus photography, OCT, ocular ultrasonography, VF, FA	/	/	Multimodal imaging of PICC.
2020 Markan [26]	Objective: To describe a case of an acquired PICC secondary to intercalary membrane detachment. Design: Case report	Description of an irido-fundal coloboma with intercalary membrane detachment associated with a ICC at the edge of a coloboma.	Hypothesis of a new pathogenic mechanism of ICC formation: the intercalary membrane detachment enables fluid from the sub-ICM to cummincate with the choroid space.	ICC secondary acquired in a case of irido-fundal coloboma.
2020 Comune [31]	Objective: To analyse the vessel density of radial peripapillary capillary in HM with (32 eyes) and without (23 eyes) PICC Design: Prospective. Examinations: OCT-A	Myopic eyes with PICC had a significantly lower vessel density than eyes without PICC, especially those with choroidal neovascularization.	/	Radial peripapillary capillary vessel density is significantly influenced (reduced) by the presence of PICC.
2021 Kim [24]	Objective: To study the choroidal microvasculature in glaucomatous eyes with PICC. Design: Retrospective Examinations: SD-OCT, OCT-A, SS-OCT, fundus examination, VF	PICC showed larger hypovascular area on En-face OCT-A 89.4% of PICC had choroidal microvascular dropout (focal sectoral capillary dropout with no visible microvascular network on deep-layers En-face images) in the area proximal to the PICC. Concordance between location of PICC and area of dropout 98% of PICC had hemifield VF defects corelating PICC hemispheric location.	Hypothesis: Common pathogenesis of PICC and microvascular dropout due to their close spatial proximity. Distortions of peripapillary tissues (due to tensile stress) induce both PICC and damage of RNFL and microvessels leading to microvascular dropout.	98% VFD corresponding to the PICC hemispheric location. Probable common pathogenic mechanisms of PICC and microvascular dropout in glaucoma. OCT-A characteristics of PICC.
2021 Liu [12]	Objective: Characterisation of PICC in Chinese highly myopic eyes and its associated risk factors. Design: Observational cross-sectional study, 890 patients with HM included. Examinations: Fundus photography, OCT	Prevalence of PICC in high myopia 3.6% (diagnosis based on the presence of typical lesion on both the fundus and the OCT). Location mainly inferior (87.5%), multiple (9.4%), superior (3.1%). Association with age, axial length and myopic spherical equivalent (based on the multiple linear logistic regression model).	Hypothesis: Impact of mechanical forces. Axial elongation during myopia progression stretches the posterior tissues leading to the appearance and progression of PICC. The lack of overlying tissues and relative thinness of the myopic conus leads to more pronounced deformation and is thus more susceptible to mechanical stress.	Prevalence of PICC in a large highly myopic population with a wide range of age (7–70 years old). PICC more frequent in eyes with severe myopic maculopathy and eyes with PS.
2021 Venkatesh [37]	Objective To compare color fundus photograph and multicolor image of PICC. Design: Case report Examinations conventional color fundus photograph, multicolor imaging, SD-OCT	Multicolor imaging seems to be less efficient than conventional color fundus photograph to diagnose PICC in myopic eyes.	/	Assessing the value of multicolor imaging in the diagnosis of PICC, the authors suggest that multicolor imaging seems to be less efficient than conventional color fundus photograph to diagnose PICC in myopic eyes.
2022 Fujimoto [38]	Objective: To evaluate 3D parameters of PICC using SS-OCT and deep learning and to correlate with VF sensitivity. Design: Retrospective Examinations: SS-OCT, deep learning	The correlation between 3D volume of PICC and VF sensitivity.	/	The 3D rendering has a potential to improve detection and pathological understanding of PICC.
2022 Ehongo [16]	Objective: To compare the peripapillary polar regions in eyes with gamma PPA and PPS in the presence or absence of PICC. Design: Observational cross-sectional study. Examinations: Fundus pictures, serial SD-OCT	PICC is a suprachoroidal detachment. PICC is aligned with the subarachnoid space. PICC is detected up on the visualisation of the ON sheaths.	Hypothesis: The pulling of ON sheaths on the scleral flange during eye movements would promote PICC.	Confirmation that PICC is as suprachoroidal detachment. Suggestion that it is promoted by tractions of the ON sheaths during eye movement.
2022 Aoki [11]	Objective: To report a case of macular lamellar hole with retinoschisis in an eye with a PICC that underwent vitrectomy with gas tamponade. Design: Case report Examinations: OCT Intervention: Vitrectomy	Non-highly myopic eye. Anatomical recovery after vitrectomy with gas tamponade Visual acuity improvement. However, a full-thickness defect at the PICC–conus junction appeared after vitrectomy.	/	Retinoschisis with lamellar macular hole may complicate a PICC even in non-highly myopic eyes.
2023 Kudsieh [39]	Objective: Assessment of the utility of OCT in the analysis of ONH and peripapillary region in highly myopic eyes with or without glaucoma. Design: Review of the literature	PICC is associated with severe myopic maculopathy and posterior staphyloma.	/	Review of the measurable ONH characteristics in highly myopic eyes, including PICC.

BM: Bruch’s membrane. BT: border tissue. ICG: indocyanine green angiography. FA: fluorescein angiography. HM: high myopia. OCT: Optical coherence tomography. SD-OCT: spectral-domain OCT. SS-OCT: swept-source OCT. OCT-A: OCT angiography. ON: optic nerve. ONH: optic nerve head. PICC: peripapillary intrachoroidal cavitation. PPA: peripapillary atrophy, PS: posterior staphyloma, RNFL: retinal nerve fiber layer. RPE: retinal pigment epithelium. TD: tilted disc. US: Ultrasound. VF: visual field. VFD: visual field defects.
